# Nanoparticle‐Based Strategies to Combat Multidrug‐Resistant Bacteria: Mechanisms, Applications, and Future Perspectives

**DOI:** 10.1002/mbo3.70318

**Published:** 2026-06-02

**Authors:** Akmal Zubair, Syeda Zaira Batool, Mohamed H. Helal, Naila Afghan

**Affiliations:** ^1^ Department of Biotechnology Quaid‐i‐Azam University Islamabad Pakistan; ^2^ Center for Scientific Research and Entrepreneurship Northern Border University Arar Saudi Arabia; ^3^ Department of Biology Kabul University Kabul Afghanistan

**Keywords:** biofilm, carbon nanotubes, clinical trials, drug delivery, gold nanoparticles, nanobiotics, silver nanoparticles

## Abstract

The infectious diseases remain a primary cause of morbidity and mortality on a global level, a crisis exacerbated by the rapid emergence of antimicrobial resistance (AMR). The ineffectiveness of the old antibiotics and the stagnation of the development of new medications have triggered an emergency in the search for new methods of treatment. This review examines the opportunity of nanotechnology, which is the so‐called nanobiotics, as a game‐changing technology in combating multidrug‐resistant (MDR) bacteria by searching various databases. Nanoparticles (NPs) are unique physicochemicals, i.e., high surface‐to‐volume ratio and multi‐valent properties that enable them to overcome traditional resistance, e.g., efflux pumps, target modifications. The paper discusses the important antibacterial processes, which can involve the induction of oxidative stress by the production of reactive oxygen species (ROS), the physical destabilization of the bacterial cell envelope, or the targeting of intracellular macromolecules, such as DNA and proteins. Moreover, it emphasizes the synergistic nature of the interactions of NPs with traditional antibiotics to improve drug delivery and efficacy and decrease host toxicity. Although a wide range of nanomaterials, such as silver, gold, metal oxides, and carbon‐based structures, promise a lot, a lot of questions have been raised on their compatibility in the long term, their impact on the environment, and their legality. Nanotechnology, coupled with precision medicine and CRISPR‐based systems, is a promising future in dealing with infectious diseases. Finally, nanomedicine is a complex solution to address the shortcomings of existing treatments and achieve a future of world health.

## Graphical abstract

Nanobiotics offer a promising strategy to combat antimicrobial resistance (AMR) and multidrug‐resistant (MDR) bacteria. Nanoparticles overcome traditional resistance mechanisms through unique physicochemical properties and multivalent interactions. Antibacterial action of nanoparticles includes ROS generation, cell membrane disruption, and intracellular targeting. Synergistic use of nanoparticles with antibiotics enhances efficacy while potentially reducing toxicity. Challenges such as long‐term safety, environmental impact, and regulatory concerns must be addressed for clinical application.

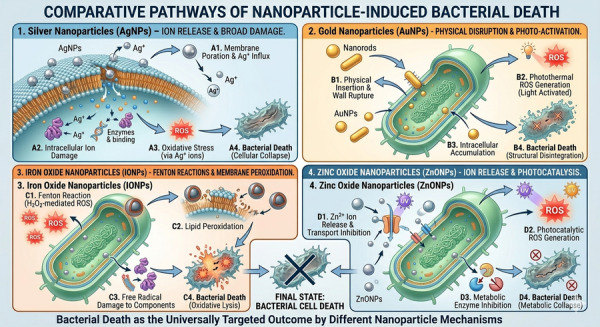

## 1 Introduction

Contemporary medicine owes its advancements to antibiotics, which have transformed the management of bacterial illnesses. The indiscriminate and widespread use of antibiotics in the past has resulted in the rapid growth of hazardous microorganisms exhibiting antimicrobial resistance (AMR). Not all bacterial strains respond to conventional treatment. Moreover, the identification of new antibiotics has not kept pace with the emergence of antimicrobial resistance. AMR accounted for 1.27 million of the 4.95 million deaths attributed to drug‐resistant microorganisms in 2019, based on data from 204 nations and territories (Mehrotra et al. 2024). As individuals with viral illnesses increasingly use antibiotics for secondary infections, the ongoing COVID‐19 pandemic is anticipated to exacerbate this trend (Ahmed et al. 2024). To combat antimicrobial resistance, innovative methods are necessary. In the fight against antimicrobial resistance, nanotechnology offers promising new opportunities. The antibacterial properties of nanoparticles are distinctive since they surpass conventional bacterial defense mechanisms. Due to their multivalency and nanoscale molecule confinement, which provide robust interactions and a high surface‐area‐to‐volume ratio, nanoparticles are mostly advantageous as therapeutic agents (Manyi‐Loh et al. 2018). Organic nanoparticles, metal oxides, nanoscale metals, and nanocomposites possess potent antibacterial properties and may be effectively used to control surface infections and infectious diseases (Jhalora and Bist 2025; Bekele et al. 2023). Diverse antibacterial nanomaterials, or nanobiotics, combat target bacteria via various mechanisms due to their unique chemical compositions and intrinsic properties. Microbial susceptibility to certain nanomaterials fluctuates significantly during the physiological life cycle, including planktonic, biofilm, stationary, nutrient‐deprived, and logarithmic growth stages (Baciu et al. 2024). Oxygen levels, pH, temperature, and several other factors influence the antibacterial efficacy of nanomaterials. The unique properties of nanobiotics facilitate the development of novel therapeutic strategies that may precisely and effectively target bacterial systems (Founou et al. 2021).

This study assesses AMR and offers a thorough evaluation of novel nanotechnologies aimed at addressing AMR, emphasizing the mechanisms of action of nanobiotics. We examine the potential of functional nanoparticles as drugs and address the issues associated with the development of novel nanobiotics.

### 1.1 Search Strategy and Selection Criteria

A thorough literature review has been carried out to find pertinent articles on the topic of nanoparticle‐based approaches to AMR. Primary electronic databases, such as PubMed, Scopus, Web of Science, and Google Scholar, were used to retrieve data. The search included publications as early as the 2000s and as recent as early 2025 to include foundational mechanical studies and more recent developments in clinical trials and nanotechnological advancements.

The search was performed using combinations of the following keywords and Boolean operators: (“nanoparticles” OR “nanobiotics”) AND (“multi‐drug resistance” OR “MDR”) AND (“antibiotics” OR “biofilm”) AND (“mechanisms” OR “targeted delivery”).

### 1.2 Inclusion and Exclusion Criteria

#### 1.2.1 Inclusion Criteria

- Original research, systematic reviews, or meta‐analyses on the antibacterial or antifungal properties of nanomaterials published in peer‐reviewed journals.
- Experiments detailing certain action mechanisms, including reactive oxygen species (ROS) production, cell wall destruction, or intracellular targeting of macromolecules.
- Clinical trials involving nanoparticle‐based interventions against infectious diseases.
- Investigations into the synergistic effects of the combination of nanoparticles with conventional antimicrobial agents.

#### 1.2.2 Exclusion Criteria

- Non‐pathogenic microbes/environmental cleanup and not related to human health.
- Did not provide a clear description of the physicochemical characteristics of the used nanomaterials (e.g., size, charge).
- Comprised abstracts that were not peer‐reviewed, gray literature, or were not in English.

The extraction and synthesis of data revolved around the healthcare burden of AMR, the effectiveness of different metallic, organic, and carbon‐based nano platforms, and regulatory and safety limitations that are impeding the advancement of widespread clinical use.

## 2 Emergence of Antimicrobial Resistance

AMR has emerged as a significant global health issue owing to the extraordinary ability of microorganisms, especially bacteria, to adapt to the selective pressures exerted by antimicrobial agents (Girma 2023; Hetta et al. 2023). AMR may develop via many means. These include alterations of antibiotic targets, active efflux of antibiotics from the cell, diminished drug entrance, and enzymatic destruction of antibiotics (Malik and Bhattacharyya 2019). Target modification denotes genetic alterations in bacteria that modify their susceptibility to antibiotics. Certain bacteria generate enzymes that may inactivate antibiotics. Horizontal gene transfer facilitates the transmission of resistance genes across bacteria using plasmids or other mobile genetic elements. Certain bacteria, fungi, and viruses generate enzymes that diminish the efficacy of antibiotics. Bacteria may actively extrude antibiotics from their cells via efflux pumps (Girma 2024a, 2024b). Modifying the permeability of the cell wall or outer membrane may diminish antibiotic infiltration. The formation of biofilms complicates therapy, since microbes might use them as a protective environment to circumvent treatment (Hetta et al. 2023). Although biofilms are mostly linked to bacteria, some harmful fungi are capable of forming them as well (Schillaci et al. 2017). The development of resistance to antimicrobial treatment in bacteria, viruses, or fungi is a multifaceted phenomenon linked to AMR. Initially, there is a decline in the number of vulnerable bacteria that have poor resistance to antimicrobial drugs (Brito 2021). The majority of susceptible microorganisms are eradicated by antimicrobial therapies upon exposure, resulting in a minimal fraction that develops resistance (Farhat et al. 2020). Antimicrobial medicines eliminate susceptible microorganisms, hence increasing the likelihood of resistant variants surviving and proliferating, particularly in conducive circumstances (Kaur and Nobile 2023). This selection pressure increases the predominance of resistant strains within the microbial population (Malinovská et al. 2023). A second crucial element of resistance propagation is the transmission of resistance characteristics. Antibiotic resistance genes may be transmitted from infected pathogens to adjacent bacteria. Every infectious agent undergoes evolution via these methods (Muteeb et al. 2023). Natural selection, horizontal gene transfer, and genetic mutations intimately combine to generate this phenomenon. Bacteria have rapidly developed and disseminated resistance mechanisms due to their genetic adaptability (Chakravarty 2020). Natural selection, horizontal gene transfer, and mutation together lead to the emergence of resistance. The emergence of drug‐resistant bacteria exacerbates the problem. The pharmaceutical industry's declining investment returns in antibiotic research and development, coupled with the insufficient production of new treatments, is exacerbating AMR (Arnold et al. 2022).

## 3 Healthcare Burden of Antimicrobial Resistance

**Figure 1:**
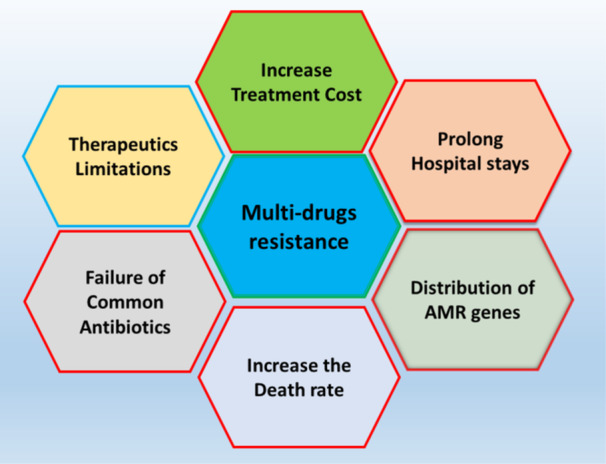
Representation of the consequences of multidrug‐resistant bacteria. AMR affects various sectors of health and life expectancy.

AMR has profound and substantial implications for public health, healthcare systems, and the economy. Significant data indicate that antimicrobial resistance adversely affects healthcare systems globally (Bassetti and Giacobbe 2020). AMR is diminishing the efficacy of conventional medical treatments, increasing the risks for those undergoing procedures such as organ transplants, chemotherapy, and surgery (Pulingam et al. 2022). Antibiotic resistance is a progressively pressing global health issue as the phenomenon continues to proliferate internationally. Immunocompromised populations, including those with cancer, SARS‐CoV‐2, or HIV, face impaired immune systems and limited therapeutic options (Zubair 2025a, 2025b, 2025a, 2025b, 2025c), and are especially susceptible to AMR as shown in Figure 1 (Gow et al. 2022).

## 4 Addressing AMR Requires Immediate Exploration of Novel and Effective Interventions

The development of innovative strategies to combat AMR is an urgent issue that must be tackled immediately. Antimicrobial resistance has been acknowledged by the World Health Organization (WHO) as a significant threat to global health (Pokharel et al. 2020). If prevailing trends persist, traditional medical treatments may pose risks, and prevalent illnesses might become untreatable. Immediate and novel strategies are essential to address AMR. A comprehensive and multidimensional approach is necessary, including healthcare antibiotic stewardship programs, CRISPR‐Cas gene editing, phage treatment, and the use of probiotics and microbiome manipulation to bolster natural defenses (Streicher 2021). Surmounting resistance necessitates the development of novel immunotherapies, the use of nanotechnology for precise drug administration, and the integration of diverse therapeutic modalities. Prioritizing vaccinations against antibiotic‐resistant illnesses is critical, and other strategies such as antimicrobial peptides and essential oils warrant investigation (Kirtane et al. 2021; Parmanik et al. 2022). Addressing AMR effectively and sustainably requires public education, a One Health framework, and global monitoring. Nanomedicine offers an innovative approach to halt the propagation of antibiotic‐resistant viruses and bacteria. This research primarily focuses on the development of nanoparticles intended for the targeted delivery of pharmaceuticals to certain organs and tissues (Weldick et al. 2022). Targeted drug administration diminishes the probability of microbial resistance by localizing treatment in the afflicted region. Nanotechnology has the potential to revolutionize the fight against infectious illnesses and enhance therapeutic effectiveness. This highlights the significance of assessing how this advanced technology might improve healthcare delivery. This publication will examine the present condition of this area and its prospective contribution to combating AMR and its effects on world health (Pulingam et al. 2022).

### 4.1 Lysis via Cell Envelope Damage

Bacterial cell walls have developed a physical defensive mechanism, being resistant to antimicrobials. Teichoic acids and lipopolysaccharides are phosphate or carboxyl groups present on the outer membranes of Gram‐positive and Gram‐negative bacteria, respectively, imparting a negative charge to the bacterial surfaces. Due to their inability to traverse membranes, hydrophobic antimicrobials have diminished efficacy against bacteria in this highly polar environment. Ultimately, inadequate energy transfer and cellular demise ensue from membrane depolarization, reduced permeability, and diminished fluidity due to electrostatic adsorption to the cell wall (Muthukrishnan et al. 2019).

Nanoparticle aggregation creates “pits” in bacterial cell walls. They may penetrate cells, change their membranes, damage structures, and kill them. Positively charged nanoparticle ions interact with bacterial surfaces’ negative charges, such as carboxyl or phosphate groups, to increase bactericidal action. Nanoparticles (NPs) cannot penetrate Gram‐positive bacterial cells due to their thick peptidoglycan covering. So far, NPs have only been found on bacteria (Wang et al. 2017). Nanoparticles (NPs) cannot penetrate Gram‐positive bacteria due to their thick peptidoglycan coating. Thus, NPs typically interact with bacterial surfaces. Antibacterial metals and oxides include silver, gold, copper, silicon, nickel, and selenium. Zinc oxide, titanium dioxide, copper oxide, magnesium oxide, and silicon dioxide are metal oxides. For their larger surface area‐to‐volume ratio, organic nanoparticles are better than inorganic ones, although their biocompatibility and biodegradability are often poor (Slavin et al. 2017). Inorganic nanoparticle cytotoxicity is dose‐dependent and depends on size and charge. Carbon nanotubes, cylindrical carbon structures with nanometer diameters, offer considerable drug delivery potential due to their chemical, physical, and biological properties. Silica mesoporous nanoparticle carriers are robust, porous, and have a huge surface area. This porous material's interior spaces enable it to hold a lot of antimicrobials (Wang et al. 2005).

### 4.2 Oxidative Stress Induction via ROS

ROS are produced by oxidative metabolism. They regulate growth, survival, and apoptosis. They also have much greater positive redox potential. ROS include hydroxyl radicals (OH), superoxide radicals (O_2_‐), singlet oxygen (O_2_), and hydrogen peroxide (H_2_O_2_). Distinct nanoparticles create distinct reactive oxygen species with varying antibacterial capabilities (Wang et al. 2017). MgO and ZnO nanoparticles only generate superoxide radicals (O_2_‐) and a mix of H_2_O_2_ and hydroxyl (OH) radicals, whereas Ag and Cu nanoparticles produce several reactive oxygen species (Fenoglio et al. 2008). Reactive oxygen species mostly originate from two sources: natural products and the disruption of the respiratory chain. A method used by scavengers to maintain minimal concentrations of these compounds is reduced glutathione (Slavin et al. 2017).

ROS are often generated and neutralized in a balanced manner within a natural environment. Under extreme stress circumstances, the formation of ROS escalates significantly, harming microorganisms and undermining the integrity of cell membranes (Quinteros et al. 2016). In the presence of oxidative stress, ROS inflict damage on intracellular macromolecules, leading to lipid peroxidation, protein alteration, enzyme inhibition, disruption of the electron transport chain, and damage to RNA or DNA. Silver (Ag), copper (Cu), zinc oxide (ZnO), titanium dioxide (TiO_2_), and iron nanoparticles (Fe NPs) are examples of nanoparticles that generate reactive oxygen species (ROS) and have potent antibacterial activity (Hailan 2022; Banerjee et al. 2022).

### 4.3 Intracellular Macromolecule Targeting (DNA, RNA, Proteins)

The functionality and survivability of bacteria rely on intracellular signaling networks and cellular homeostasis. Cell death can occur when nanomaterials are altered to impede specific pathways. Errors in gene expression, DNA damage, and protein synthesis exemplify disturbances (Chatterjee et al. 2014; Shamaila et al. 2016). An instance is the synthesis of pyrimidine‐capped AuNPs (Au‐DAPT), achieved by incorporating 4,6‐diamino‐2‐pyrimidinethiol (DAPT), a derivative of 2‐pyrimidinethiol present in E. coli, into AuNPs. These nanoparticles inhibit the proliferation of drug‐resistant microorganisms, including *Pseudomonas aeruginosa* and *Escherichia coli* (Zhao et al. 2010).

### 4.4 Biofilm Structural Degradation

Nanoparticles may disrupt biofilm owing to their exceptional penetrating capacity. Upon traversing the extracellular polysaccharide matrix of the biofilm, they initiate bactericidal interactions with the bacteria. The interaction and penetration of nanoparticles inside biofilm are strongly affected by their charge. Cationic nanoparticles, for instance, exhibit significant interactions with anionic matrices due to their positive charge (Sutherland 2001; Hosnedlova et al. 2022).

## 5 Synergistic Effects of Nanoantibiotics

The effectiveness of nanoparticles may be significantly enhanced by coating or integration with other substances. The amalgamation of nanoparticles with antibiotics may significantly diminish bacterial resistance. Antibiotics, whether combined with or incorporated into nanoparticles, provide resistance to the enzymes and chemicals that would typically break them. This barrier may enhance a drug's therapeutic efficacy. A dosage decrease is essential to enhance treatment effectiveness and minimize host risk (Brown et al. 2012; Li et al. 2019; Abo‐Shama 2020). By using treatments that activate various mechanisms and focusing on cargo release, bacteria are safeguarded against sub‐minimal inhibitory concentrations of the medication, while nano‐carriers facilitate the penetration of antibiotics through bacterial cell membranes (Bera and Mondal 2018). This thus reduces resistance selection. The antibiotic‐induced degradation of the cell membrane markedly facilitates the ingress of nanoparticles and their complexes into the body.

The combination of amoxicillin with AgNPs significantly reduces bacterial growth, whereas the AgNPs‐ampicillin pairing has greater efficacy against *Staphylococcus, Escherichia coli*, and *Klebsiella*, as shown by prior research. Abdelghany et al. (2012) demonstrates enhanced effectiveness against *P. aeruginosa* in both in vitro and in vivo environments when gentamicin is encapsulated in poly (lactide‐co‐glycolide) (PLGA) nanoparticles; this advantage is further emphasized in comparison to the administration of amoxicillin alone (Abdelghany et al. 2012). The creation of broad‐spectrum bactericidal agents that bypass the resistance mechanisms of multidrug‐resistant strains of P. aeruginosa, *Enterobacter aerogenes*, and Methicillin‐resistant *Staphylococcus aureus* (MRSA) was facilitated by the conjugation of ampicillin to the surfaces of AuNPs and AgNPs (Li et al. 2005). Bimetallic nanoparticles combined with core‐shell linezolid exhibited potent bactericidal activity against all studied species, including MRSA, whereas colistin showed increased effectiveness against antibiotic‐resistant *E. coli* (Chamundeeswari et al. 2010). Moreover, *E. coli* may be mitigated by combining it with alginate nanoparticles and other compounds such as polyamines, lactic acid, and components of essential oils (Hazime et al. 2022).

## 6 Metallic Nanomaterials as Antibacterial Agents

Numerous ideas and research indicate that nanoparticles (NPs) may alter the morphology and structure of microorganisms, hence incapacitating their survival. Sondi and Salopek‐Sondi (2004) illustrated the phenomenon of nanoparticles adhering to bacterial cell walls or membranes, infiltrating them, and inducing cell death via structural disruptions in membrane permeability (Sondi and Salopek‐Sondi 2004). Nanometers are the smallest unit of measurement for nanomaterials, underscoring their very minuscule dimensions. Minuscule in size, they exhibit significant differences from bulk materials in some critical physicochemical aspects (Rosi and Mirkin 2005). Lee et al. (2019) assert that these characteristics dictate their antibacterial efficacy, drug transporter utility, and toxicity (Lee et al. 2019). Nanoparticles, ranging in size from 1 to 100 nm, may engage with many biomolecules inside cells, including DNA, proteins, and lipid membranes (Nel et al. 2009). Interactions may damage bacterial DNA and cell membranes (Aljuffali et al. 2015). Research by Morones et al. (2005) indicates that silver nanoparticles (Ag NPs) sized between 1 and 100 nm have enhanced bactericidal efficacy against gram‐negative bacteria (Morones et al. 2005). This is due to the nanoparticles' capacity to establish close contact with bacterial cells. Their small size should enable them to overcome any biological impediment. Their size may influence their pharmacokinetic characteristics (Hoshyar et al. 2016).

## 7 Silver Nanoparticles (AgNPs) Against AMR Bacteria

**Figure 2:**
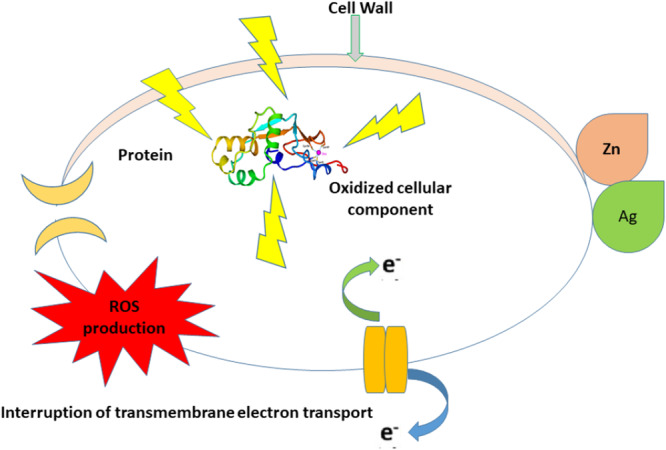
Schematic illustration of antimicrobial actions of Zn/Ag nanoparticles, including disruption of the cell wall, oxidative damage to cellular proteins, and interruption of transmembrane electron transport leading to reactive oxygen species (ROS) generation. These combined effects result in oxidation of cellular components and loss of microbial viability.

AgNPs are the most sought‐after commercial nanoparticles and may lead to a new class of medicines with robust antibacterial characteristics. They are safer than other nanoparticles, and antibacterial research has evolved swiftly. Singh (2015) stated that AgNPs damage bacteria by adhering and infiltrating their cell membranes (Figure 2) (Singh 2015).

When Ag+ ions damage interior structures, ROS and oxidative stress result (Singh 2015; Flores‐López et al. 2019). Several signal transduction pathways, including the sodium/potassium ATPase pump, are affected. Ag+ ions and AgNPs with phosphorus‐containing DNA may inactivate proteins and kill bacteria (Maurer and Meyer 2016). They found that interactions between oxygen, sulfur, chlorine, and thiols may significantly affect the release of Ag+ ions. According to Sriram et al. (2012) (Sriram et al. 2012) and Abuayyash et al. (2018) (Abuayyash et al. 2018), dimensions greatly affect Ag+ ion emission.

**Figure 3:**
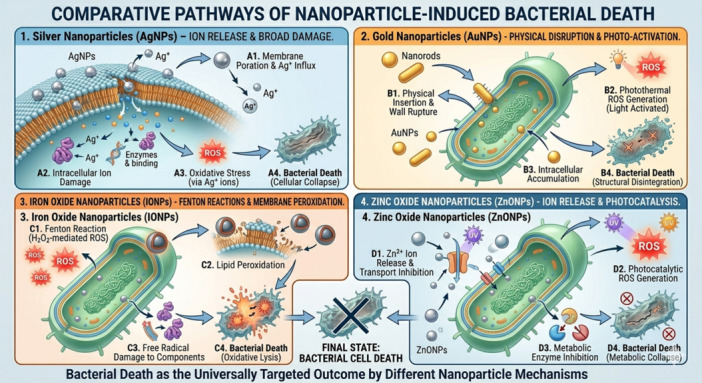
A comparative overview of nanoparticle antibacterial strategies, illustrating how different types, AgNPs, AuNPs, IONPs, and ZnONPs, utilize distinct physical and chemical pathways to disrupt vital bacterial functions and induce cell collapse.

Because of their small size, AgNPs may enter cells quickly. They cause cell death and increase membrane permeability by changing their shape and composition. AgNPs work depending on the bacterium. Differences in cell wall construction, thickness, and content cause this (Tamayo et al. 2014). The production of reactive oxygen species and breakdown of the cell wall and plasma membrane give AgNPs their unique antibacterial properties, as shown in Figure 3 (Labay et al. 2019).

## 8 Gold Nanoparticles (AuNPs) for Antimicrobial Applications

AuNPs have garnered academic attention. Abdel‐Raouf et al. (2017) say AuNPs may be spherical, triangular, hexagonal, or rod‐like (Abdel‐Raouf et al. 2017). Triangular‐shaped AuNPs exhibited superior antibacterial efficacy against several microbes compared to spherical‐shaped AuNPs. They modify membrane potential and diminish ATP synthase enzyme activity, among other impacts on bacterial metabolism. Like AgNPs, AuNPs may jeopardize the stability and structural integrity of cell membranes (Rattanata 2016). Cui et al. (2012) assert that AuNPs inhibit the binding of rRNA to its subunit, hence obstructing protein translation. Gold nanoparticles (AuNPs) may bind to nucleotides containing phosphorus or sulfur. The potent antibacterial effectiveness of antibiotics is often enhanced when included with AuNPs (Cui et al. 2012). Brown et al. (2012) conducted a study that showed the effectiveness of ampicillin in conjunction with AuNPs against ampicillin‐resistant *E. coli, Enterobacter aerogenes*, methicillin‐resistant *Staphylococcus aureus*, and *Pseudomonas aeruginosa* (Brown et al. 2012). The AuNPs‐AMP combination catalyzes the transmembrane pump while blocking and disrupting drug efflux. The significant quantity of β‐lactamase generated by bacteria is effectively neutralized by the amalgamation of AuNPs and AMP.

## 9 NO‐Delivery Nano Platforms Against Antimicrobial Resistance

**Figure 4:**
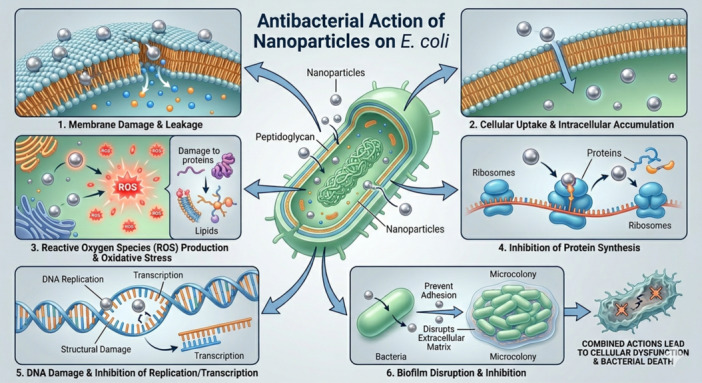
Schematic representation of the multifaceted antibacterial mechanisms of nanoparticles, including membrane disruption, intracellular penetration, oxidative stress via ROS generation, interference with protein synthesis and genetic processes, and inhibition of biofilm formation. These combined actions ultimately lead to cellular dysfunction and bacterial cell death.

Various susceptible and antibiotic‐resistant bacteria, including *E. faecalis, E. coli, K. pneumoniae, S. pyogenes*, and *P. aeruginosa*, exhibit substantial growth inhibition when exposed to nitric oxide (NO) releasing nanoparticles (NONPs). Natural gas, or NO, has traits such as hydrophilicity, lipophilicity, and oxygen instability (Fang 1997). Nitric oxide (NO) eliminates microorganisms and induces cellular damage via its reactive nitrogen and oxygen species. They are the result of the reaction between nitric oxide and superoxide or oxygen. The production of these intermediates is physiologically significant at NO concentrations above 1 M. Reactive nitrogen species (RNOS), including peroxynitrite, nitrogen dioxide, iron‐dinitrosyl complexes, and S‐nitrosothiols (RSNO), are generated at these concentrations, as seen in Figure 4 (Fang 1997).

Rubbo et al. (1994) revealed that nitric oxide degrades lipids via nitrogen dioxide and peroxynitrite (Rubbo et al. 1994). Peroxynitrite‐induced lipid peroxidation in liposomes enhances the antibacterial properties of nitric oxide (Deupree and Schoenfisch 2009). Reactive thiols, heme groups, aromatic or phenolic amino acids, amines, and tyrosyl residues are among the inert chemical groups and residues found in proteins (Rong et al. 2019). Researchers have shown that nitric oxide radicals may inactivate aconitase, NADH dehydrogenase, and succinate dehydrogenase in studies of nitric oxide‐induced cytotoxicity. This offers more evidence that nitric oxide radicals may induce iron shortage by directly releasing iron from metalloenzymes (Drapier et al. 1991). Nitrosating intermediates induce DNA damage when reactive nitrogen species (RNOS) autoxidize nitric oxide, leading to the deamination of cytosine, adenine, and guanine. Nitrogen dioxide and peroxynitrite are the most potent DNA regulators and disruptors among these reactive nitrogen species (RNS). Cysteine residues in DNA alkyl transferases may interact with nitric oxide to form NO‐S adducts owing to the presence of ‐SH groups. This occurs because these adducts inhibit the protein from acquiring the guanine alkyl group. Nitric oxide (NO) impedes the functionality of DNA repair enzymes on alkylated DNA, rendering such DNA irreparable (Laval and Wink 1994; Jena 2012). Prokaryotes have a greater reliance on iron‐sulfur clusters than human cells, rendering them more susceptible to nitric oxide treatment (Fang 2011).

## 10 Cu‐ and CuO‐Mediated Antimicrobial Nanostrategies

El‐Batal et al. (2017) and Asemani and Anarjan (2019) have shown the antibacterial properties of copper and its oxide nanoparticles (CuNPs or CuONPs) (El‐Batal et al. 2017; Asemani and Anarjan 2019). The negative charge of nanoparticles (NPs) contributes to their antibacterial characteristics, as shown by the current research (Bogdanović et al. 2014). The Cu2+ ion may penetrate the cell by bypassing the lipid bilayer. Reactive oxygen species (ROS) are rapidly released upon cellular entry. Lipid oxidation and protein oxidation are interrelated processes (DeAlba‐Montero et al. 2017). The antibacterial properties of copper are evident as its oxidation state transitions from +1 to +2. Research indicates that the antibacterial efficacy of CuONPs may be significantly influenced by their combination with amino acids (Badetti et al. 2019).

## 11 ZnO Nanoparticle‐Based Antibacterial Systems

Bhuyan et al. (2015) assert that zinc oxide nanoparticles possess exceptional antibacterial properties. Previous research indicated that the surface coating of ZnONPs may impede their interaction with biological fluids (Bhuyan et al. 2015). Agglomeration results from the high affinity and interactions of PEGylated ZnONPs with lactic and citric acid. Pranjali et al. (2019) found that ZnONPs coated with PEG exhibited significantly reduced bacterial inhibitory efficiency compared to their uncoated counterparts. Upon interaction with a bacterial cell, ZnONPs compromise the bacterial cell membrane and release zinc ions (Pranjali et al. 2019). The interaction of zinc ions with several intracellular components leads to increased cellular damage (Li et al. 2011). The formation of reactive oxygen species may be facilitated by zinc ions (Kumar et al. 2011; Singh et al. 2020). According to a study, thiosemicarbazide and glutamic acid‐functionalized ZnONPs influenced the expression of efflux pump genes in a variety of drug‐resistant S. aureus strains (Nejabatdoust et al. 2019).

## 12 Titanium Dioxide Nanoparticles (Tio_2_ NPs)

Singh et al. (2018) assert that TiO_2_ nanoparticles induce oxidative phosphorylation disruption and cellular membrane impairment via the generation of reactive oxygen species (ROS). TiO_2_NPs not only impede the coenzyme‐independent respiratory network and signaling pathways but they also inhibit the absorption and transport of iron and phosphorus (Singh et al. 2018). Foster et al. (2011) discovered that it also diminishes the production and degradation of heme groups (Foster et al. 2011). Research has shown its capacity to produce free radicals, as well as being reliant on light (Wu et al. 2010). Liu et al. (2010) exhibited their ability to disrupt peptidoglycans, lipopolysaccharides, and phospholipid bilayers (Liu et al. 2010).

## 13 MgO Nanoparticle‐Based Antibacterial Systems

ROS produced by MgONPs are posited to induce significant cellular damage (Krishnamoorthy et al. 2012; He et al. 2016). Nguyen et al. assert that magnesium oxide nanoparticles (MgONPs) disrupt E. coli cell membranes and inhibit the formation of S. epidermidis biofilms. They may suppress bacterial growth by quorum sensing, ROS generation, or the modulation of calcium ion (Ca2 + ) concentrations. Nguyen et al. (2018) assert that the adhesion of MgONPs to cell membranes compromises their integrity, resulting in the leakage of intracellular components. Magnesium oxide and manganese oxide nanoparticles were synthesized using an extract from *Matricaria chamomilla L* (Nguyen et al. 2018). The data indicate that nanoparticles may penetrate cells and compromise their membranes. This resulted in the release of cytoplasmic material from inside the cell, and research indicates that MgONPs are effective against bacteria due to their generation of Mg2+ ions, interaction with the cell membrane, and alteration of pH, as seen in Table 1 (Ogunyemi et al. 2019).

**Table 1:** Class of nanoparticles and their mode of action in the targeted organism.

Nanoparticles (NPs)	Minimum concentration	Mode of action	Target microorganisms	In Vitro Model/Cell Line	References
CuO NPs	1–10 μg/mL	ROS generation	*Streptococcus mutans, Lacticaseibacillus casei, Lactobacillus acidophilus*	Human Gingival Fibroblasts (HGF)	(Amiri et al. (2017))
	100–1,000 μg/mL	ROS generation	*Candida albicans, C. glabrata, C. krusei*		(Amiri et al. (2017))
Green‐synthesized ZnO NPs	0.025 mg/mL	Breakdown of membranes and production of ROS	*Escherichia coli, Staphylococcus aureus, Klebsiella pneumoniae, Enterococcus faecalis*	HaCaT (Human Keratinocytes)	(Demissie et al. (2020))
SPIONs (Superparamagnetic Iron Oxide NPs)	20 μg/mL	—	*Pseudomonas aeruginosa, Candida albicans*	L929 (Mouse Fibroblasts)	(Antony (2020))
AgNPs	4–16 μg/mL	Scavenging of hydrogen peroxide	*Escherichia coli, Enterococcus faecalis, Salmonella typhi*	HEK‐293 (Human Embryonic Kidney	(Keshari et al. (2020))
Green‐synthesized nano copper	—	Membrane disruption; ROS‐induced oxidative stress	*E. coli, Proteus sp., Enterococcus sp., Klebsiella sp*.		(Wu et al. (2020))
SeNPs	82–660 μg/mL	Membrane breakdown and oxidative stress	*Escherichia coli, Staphylococcus aureus*	HepG2 (Human Liver Cancer)	(Vahdati and Tohidi Moghadam (2020))
AuNPs/MOFs hybrid	—	Peroxidase‐like activity; production of hydroxyl radicals (•OH)	*Escherichia coli*,	NIH/3T3 (Mouse Embryo Fibroblasts)	(Hu et al. (2021))
TiO_2_ NPs	1000 μg/mL	ROS generation	*Escherichia coli*	—	(Karahan et al. (2018))
AgNPs	100 μg/mL	Membrane disruption	*Ralstoniasolanacearum, Meloidogyne*	—	(Khan et al. (2021))
Garcinia NPs + MV irradiation	4 mg/mL	Outer membrane disruption	*Escherichia coli*	—	(Qiao et al. (2022))
Nickel oxide NPs	20 μg/mL	Ni^2+^ ion release; ROS generation	*Escherichia coli*	HeLa (Human Cervical Cancer)	(Al‐Zaqri (2022))
ZnO NPs	0.2–1.4 mM	FtsZ interference, membrane rupture, and electrostatic interaction	*Pseudomonas aeruginosa, Staphylococcus aureus*,	In Vivo (Mouse Skin)/HaCaT	(Mendes et al. (2022))
PVA aerogel microspheres with biogenic ZnO NPs	12.5 U/mL	Zn^2+^ ion release; ROS production	*Klebsiella pneumoniae, Pseudomonas aeruginosa, Escherichia coli*	Human Skin Fibroblasts	(Abdul Malek et al. (2025))
PVA/starch/chitosan films with NiO–CuO	160–0.156 μg/mL	ROS production and metal ion release	*Escherichia coli, Staphylococcus aureus*	Primary Human Fibroblasts	(Momtaz et al. (2024))
TiO_2_ nanorods	10 mM	Ag^+^ ion release; ROS production	*Staphylococcus aureus*	A549 (Human Lung Epithelial)	(Korcoban et al. (2025))

## 14 Engineered Iron Oxide Nanostructures (Fe_3_O_4_ NPs)

Research indicates that ferric oxide nanoparticles may diminish H + efflux across microbial cell membranes. Iron oxide, when delivered as nanoparticles, markedly reduces ATP‐related metabolism. A reduction in membranes associated with H2 production was observed by Gabrielyan et al. (2019). The antibacterial efficacy of iron oxide nanoparticles (Fe_2_O_3_NPs) and biosynthesized iron nanoparticles (Fe NPs) has been thoroughly investigated (Sathishkumar et al. 2018; Madivoli et al. 2019). Numerous studies have examined the antibacterial properties of calcium oxide nanoparticles (Butt 2015; Balaganesh 2018).

## 15 Dendrimers as Antimicrobial Nanocarriers

Dendrimers are a class of highly branched, tree‐like macromolecules with diverse biological activities, which may be classified according to their fundamental structure and surface properties. Extremely high percentage (97.98%). A plethora of research has concentrated on polyamidoamine (PAMAM) dendrimers due to their drug‐encapsulating and conjugating capabilities, in addition to their uniform structure (Chis et al. 2020; Svenson and Tomalia 2005). Cationic and higher‐generation PAMAM dendrimers demonstrate increased immunogenicity and cytotoxicity compared to anionic or neutral PAMAM dendrimers. Meticulous design is essential to ensure the safe medical use of PPI dendrimers, given their bioactivity and extensive functionalization options, due to the toxicity linked to their production and terminal group chemistry. Due to their silicon‐based structures, Carbosilane dendrimers exhibit lower immunogenicity and toxicity compared to cationic PAMAM dendrimers, making them more suitable for biological applications (Prakash 2007). Dendrimers composed of peptides are optimal for gene transfer and antimicrobial applications due to their design, which ensures minimum immunological reactivity and high biocompatibility, while preserving diverse functionalities. The administration of antimicrobial medications was among the first uses of dendrimers as pharmacological carriers (Tomalia and Fréchet 2001). Early investigations showed their ability to target medication release and load drugs against resistant microorganisms. Dendrimers' 125 surface functionalities determine their bioactivity, which includes membrane penetration and drug delivery. Neutral or anionic terminal groups minimize cytotoxicity and immunogenicity, although cationic dendrimers may cause cell death by disrupting electrostatic membranes. Despite their potential, dendrimers have significant limitations (Malik 1999). Cytotoxicity (especially in cationic forms), immunogenicity, diminishing permeability with generations, and complex or expensive purification and production are some of the downsides. Biological dendrimers undergo several changes to enhance efficiency and safety, including surface neutralization, which reduces immunogenicity, and PEGylation, which increases solubility and circulation (Fateh et al. 2024; Li et al. 2023). We must evaluate dendrimers' categorization, production, and functionalization to make them more consistent for diagnostic or therapeutic use, with fewer side effects, and more biocompatibility (Malik 1999). Dendrimer production may make insoluble medicines more accessible and soluble. Particles that react to pH and enzyme levels may be targeted for delivery and treatment of disorders resistant to traditional therapy. As indicated in Table 2, high‐generation dendrimers are too complex and poisonous for practical usage (Kesharwani 2021).

**Table 2:** Multidrug‐resistant bacteria and the use of nanoparticles for their eliminations along with the mechanism of action.

ype of nanoparticles	Targeted bacteria	Antibiotic resistance type	Mechanisms of antibacterial action	References
**AgNPs**	*Enterococcus faecalis, S. aureus*	Vancomycin‐resistant	Vancomycin and the synergistic effect that results in cell death	(Saeb et al. (2014); Esmaeillou et al. (2017))
	*Enterococcus spp*.	—	Ongoing investigations	(Percival et al. (2007))
	*E. coli, S. aureus*	Tetracycline‐resistant	Tetracycline synergy	(Djafari et al. (2016))
	*P. aeruginosa*	Ofloxacin‐resistant	Circumventing multidrug efflux pumps	(Ding et al. (2018))
	*E. coli*	MDR	ROS generation	(Zhang et al. (2013))
	*E. coli, P. aeruginosa*	—	—	(Ramalingam et al. (2016))
	*S. aureus, Enterococcus spp., P. aeruginosa, A. baumannii, Enterobacteriaceae*	—	Interaction with biological components affecting chemical and physical qualities	(Cavassin et al. (2015))
	*E. coli*	—	—	(Lok et al. (2007))
	*S. aureus, E. coli, P. aeruginosa, K. pneumoniae, B. subtilis*	—	Bacterial cell wall penetration	(Acharya et al. (2018))
	*P. aeruginosa*	—	Blue light‐based synergistic treatment	(Din (2016))
	*E. coli, K. pneumoniae*	Cefotaxime‐resistant	DNA damage and disintegration of cell walls	(Shaikh et al. (2017))
	*S. aureus, E. coli, P. aeruginosa, E. aerogenes*	Ampicillin‐resistant	Combining ampicillin to facilitate cell entrance	(Brown et al. (2012))
	*Multiple species*	Kanamycin‐resistant	Disruption of the cell wall	(Payne et al. (2016))
	*Multiple MDR*	—	ROS production, membrane disruption, and photothermal/photodynamic treatments	(Shaker and Shaaban (2017))
**zZnONPs**	*K. pneumoniae*	Ampicillin/carbenicillin‐resistant	ROS production and rupture of membranes	(Reddy et al. (2014))
	*S. aureus*	MRSA	Inhibition of enzymes	(Cha et al. (2015))
	*E. coli*	MDR	ROS‐mediated membrane disruption	(Li et al. (2012))
	*S.aureus,P. aeruginosa*	Biofilm	ROS production	(Aswathanarayan and Vittal (2017))
**CuO NPs/CuNPs**	*E. coli, S. aureus*	MDR	ROS production	(Singh et al. (2014))
	*S. aureus*	MRSA	Cu2 + ‐DNA binding leading to helical disruption	(Kruk et al. (2015))
	*P. aeruginosa*	Biofilm	Penetration of cell walls and damage to nucleic acids	(LewisOscar et al. (2015))
**Fe_3_O_4_ NPs**	*E. coli*	MDR	RF‐induced disruption of the membrane	(Chaurasia et al. (2016))
	*S. aureus, P. aeruginosa, E. coli*	—	Membrane penetration; electron transfer interference	(El Zowalaty et al. (2015))
**Al_2_O_3_ NPs**	*S. aureus*	MRSA	Breakdown of cell walls and production of ROS	(Ansari et al. (2013))
	*E. coli*	MDR	Intracellular accumulation	(Ansari et al. (2013))
**TiO_2_ NPs**	*S. aureus*	MRSA	Thiol protein binding and ion release	(S. Roy et al. (2010))
	*E. coli*	MDR	ROS and rupture of membranes	(Li et al. (2012))
**Bimetallic NPs**	*S. aureus*	MRSA	DNA damage, disruption of membranes, and suppression of protein synthesis	(Foster et al. (2011))
Au/Ag NPs	*Enterococcus spp*.	Vancomycin‐resistant	Antimicrobial PDT plus SERS	(Zhou et al. (2018))
	Multiple biofilm‐forming bacteria	—	Disruption of cell walls and inactivation of enzymes	(Ramasamy et al. (2016))
Au/Pt NPs	*E. coli*	MDR	Damage to the inner membrane	(Zhou et al. (2018))
Cu/Ni NPs	*S. aureus, E. coli, S. mutans*	MDR	ATP increases ion adsorption	(Argueta‐Figueroa et al. (2014))
**Graphene Oxide NPs**	*S. aureus*	MRSA	Antibiotic combination + NIR exposure	(Pan et al. (2016))
GO NPs	*E. coli, E. faecalis*	MDR	UV‐driven ROS	(Govindaraju et al. (2016))
**SeNPs**	*S. aureus, E. coli*	MDR	Cell wall disruption; targeted treatment	(Huang et al. (2017))
	*S. aureus*	MRSA	NIR‐directed photothermal treatment	(Zhao et al. (2014))

## 16 Carbon Nanostructures as Antibacterial Agents

Many carbon‐based nanostructures with biological uses and antibacterial qualities include graphene quantum dots (GQDs), fullerenes, carbon nanotubes (CNTs), graphene oxide (GO), reduced graphene oxide (rGO), carbon nanofibers, and carbon dots (CDs). Top‐down (chemical synthesis or vapor deposition) and bottom‐up (graphite exfoliation) processes may manufacture graphene, graphene oxide (GO), and carbon nanotubes (CNTs) (Fritea et al. 2021; Maleki Dizaj et al. 2015). Carbon‐based nanomaterials like graphene oxide and carbon nanotubes can disrupt microbial membranes and generate oxidative stress to improve physical and chemical functionalization, targeting, drug loading, and dispersibility, according to research by Azizi‐Lalabadi et al. (2020). Heteroatom doping, bioconjugation, noncovalent interactions, and functional group covalent attachment are examples. Surface chemistry, particle size, and functionalization greatly affect carbon‐based nanostructure immunogenicity and cytotoxicity (Du et al. 2023; Laganà et al. 2023). A recent study shows that functionalization improves biocompatibility, reduces immunogenicity, and customizes bioactivity. In many biological situations, aggregation, sharp edges (like GO sheets), and residual catalysts that may induce cytotoxicity or immunological activation are detrimental (Kharlamova and Kramberger 2023; Liu et al. 2011).

Several antimicrobial mechanisms have been discovered. When carbon nanotubes (CNTs), graphene oxide (GO), or fullerenes touch bacterial cell walls, they may slow metabolism and kill cells. This includes an excess of reactive oxygen species (ROS), interference with energy metabolism or DNA activity, and mechanical membrane breakage (particularly for two‐dimensional structures like GO) (Fritea et al. 2021).

Size and surface charge determine carbon nanostructures’ antibacterial properties. Smaller particles interact better with bacteria due to their larger surface area to volume ratio. Positively charged surfaces may stick to negatively charged bacterial membranes, increasing membrane rupture risk. Studies show that smaller carbon dots kill bacteria better. This is attributed to improved membrane contact and ROS production (Maleki Dizaj et al. 2015). Antibacterial mechanisms may be achieved on carbon‐based nanoplatforms. Fullerenes and graphene oxide (GO) operate via membrane slicing and oxidative stress, carbon dots and photoinduced processes, virgin or functionalized carbon nanotubes and nanodots, and hybrid composites, which generally produce ROS and disturb metabolism (Kang et al. 2008).

The immunologic profile, therapeutic application, and antibacterial activity of carbon nanomaterials depend on their kind, size, surface charge, functionalization, and hybridization. There are several types of GO nanostructures, including nanosheets, quantum dots, nanoribbons, and hybrid composites with other materials, including metal nanoparticles or polymeric matrices (Zarouki et al. 2025).

Chemical exfoliation, Hummers' method, and electrochemical methods are used to synthesize structures' physical and chemical characteristics. Layer count, lateral dimension, oxidation level, and defect density affect antibacterial efficiency. GO nanostructures may conjugate metals (e.g., silver, nickel, copper), polymers, and proteins, and non‐covalent adsorption and covalent grafting can further functionalize them (Raul et al. 2022). Functionalization techniques improve GO's bioactivity and stability, affecting its therapeutic potential, immunogenicity, cytotoxicity, and safety by 16, 14, 12, and 14%, respectively. Some drawbacks of using GO nanostructures include their inherent size and compositional variation, the possibility of aggregation, the difficulty of regulating toxicity, and the difficulty of large‐scale synthesis. (16%), (14%), 12%, (14%) (Mohammed et al. 2020). Size (smaller sheets may damage membranes and increase oxidative stress), surface charge (typically negative but variable), and oxidation level are important features of GO. Larger sheets trap bacteria. Drugs and sheets with sharp edges and high defect density may pierce bacterial membranes and wrap or entrap microorganisms. (16%), (14%), 12%, (14%). Adding AgNPs, copper nanoparticles, and nickel colloidal clusters to GO nanoplatforms may help them fight bacteria and other resistant pathogens. The precise GO‐based nanostructure determines how these nanoplatforms release metal ions from the composite, trap, and perturb metabolism, or shatter membranes with sharp nanosheets. Functionalization with AgNPs increases bactericidal action via physical and chemical processes, whereas hybridization with biomolecules or polymers reduces cytotoxicity and improves biocompatibility (Tene et al. 2024; Du et al. 2022).

The study suggests that graphene oxide may fight Klebsiella and methicillin‐resistant Staphylococcus aureus. Its synergistic properties with antibiotics and other nanoparticles make it effective in nanocomposites. Its biocompatibility and water dispersibility enable drug delivery, wound dressings, and biofilm clearance, among other applications (Ravikumar et al. 2022; Shankar et al. 2023; Itoo et al. 2022).

## 17 Carbon Nanotube and Fullerenes as Antimicrobial Nanomaterials

Each CNT nanostructure has its own structure and manufacturing technique. Some carbon nanotubes have single, multiple, or double walls. Geographical multi‐walled carbon nanotubes (MWCNTs) typically have several concentric graphene cylinders and are made using gas‐phase deposition, laser irradiation removal, or arc evaporation, unlike SWCNTs, which are made using other methods. Covalently attaching proteins, polymers, or medicines to carbon nanotubes (CNTs) improves their efficacy. This improves antibacterial efficacy, solubility, dispersion, and cytotoxicity (Karaky 2020; Saleemi et al. 2022). Needle‐shaped CNTs with improved electrical conductivity and structural strength may permeate bacterial cell membranes and kill cells by releasing cytoplasm. Carbon‐based nanomaterials, single‐tube or multi‐tube, may have antibacterial characteristics when coupled with metal ions (Vardharajula 2012; Mohammadi et al. 2020). This would position them as a prospective antibiotic delivery mechanism capable of circumventing efflux pumps. They provide crucial assistance for biosensors and facilitate medication delivery. Carbon nanotubes have the ability to traverse cell membranes, potentially disrupting many biological functions (Mousavi et al. 2022).

The surface chemistry, physical properties, and extent of functionalization of carbon nanotubes are intimately correlated with their immunogenicity and bioactivity (Teixeira‐Santos et al. 2021). Unlike pristine carbon nanotubes (CNTs), which can induce cytotoxicity and inflammatory reactions despite their notable bioactivity, functionalized CNTs—especially those modified with carboxyl or amino groups—typically demonstrate reduced toxicity and enhanced biocompatibility. Common adverse effects include edema, oxidative stress, aggregation in biological environments, and batch heterogeneity, resulting from variations in surface properties and synthesis methods (Liu et al. 2018; Abo‐Neima et al. 2020).

Their antibacterial efficacy is considerably influenced by their intrinsic physicochemical properties, such as diameter, surface charge, and length. Smaller SWCNTs, with a greater surface area, are expected to exhibit enhanced antibacterial properties owing to their ability to penetrate the bacterial envelope and induce the efflux of internal cellular constituents. This results from their increased tendency to engage with and destroy bacterial cell envelopes. 0.13 multiplied by 127 equals The length of carbon nanotubes (CNTs) affects their toxicological properties. Shorter tubes have heightened open‐end density, hence augmenting their antibacterial properties, while longer tubes tend to be less detrimental to bacteria (Liu et al. 2018). Surface charge decreases membrane potential, increases cell adhesion, and modulates electrostatic interactions with negatively charged bacterial membranes, affecting antibacterial effectiveness. CNT nanoplatforms may be made from drug‐conjugated CNTs, metal oxide‐functionalized CNTs, polymer‐CNT composites, and pure CNTs. These platforms disrupt membranes, produce ROS, encapsulate bacteria, and impede metabolic activities. Due to electron transport and reactive oxygen species generation, metallic carbon nanotubes exhibit better antibacterial activity than semiconducting ones. The antibiotic effect may be enhanced by light activation, which enhances ROS production in bacteria (Hassani et al. 2022). The range, effectiveness, and safety of CNT‐based antibacterial nanostructures depend on four factors: CNT type, synthesis and functionalization, physicochemical properties, and platform material (Hou et al. 2022).

Amino‐functionalized fullerenes belong to the class of functionalized fullerenes. Some fullerenes, including C60 (buckminsterfullerene) and C70, have metal atoms or clusters. Fullerenes are usually made by arc discharge, chemical vapor deposition, or laser ablation. Bioactivity, solubility, and ligand incorporation may be changed by adding amino, carboxyl, or hydroxyl groups (Bolshakova et al. 2023). Functionalization changes their biological interactions, hydrophobicity, immunogenicity, biocompatibility, and antibacterial and cytotoxic properties. Fullerenes and their derivatives have many biological constraints in the 135–137 nm area. Drug aggregation, surface chemistry, and dosage determine cytotoxicity or inflammation (Nimibofa et al. 2018). Antibacterial activity is significantly influenced by particle size and surface. Components. Because small fullerenes have higher surface areas, they can adsorb onto bacterial membranes and enter bacterial cells better as bactericides. Cationic fullerene derivatives electrostatically interact with Gram‐negative bacteria, disintegrating and killing cell membranes. Gram‐negative bacteria's surfaces are negatively charged. The ineffectiveness of anionic or neutral fullerenes follows the same principle.

Heredia et al. (2022); Cheng et al. (2025). Fullerene nanoplatforms differ in design and function. Microorganisms primarily suffer oxidative, membrane, protein, and DNA strand damage from pure fullerene irradiation. ROS are released into the air. Light‐induced and antioxidant effects were also observed in fullerene C60 nanostructures. Their complex, wriggly structure absorbs oxygen molecules and releases reactive oxygen species when exposed to light. They help give medicines more accurately while destroying bacteria via oxidative stress. Photodynamic approaches employing derivatives of hydrophilic or peptide‐attached fullerene, which promote membrane contact and reactive oxygen species, may target many bacteria, including drug‐resistant types. Despite their molecular bonding, fullerene nanoparticles are more antimicrobial. These include decreased bacterial energy metabolism and improved medication penetration. Fullerenes on metal, peptide, and polymer nanoplatforms may improve pathogen coverage and synergistic antibacterial actions (Javed Ansari et al. 2022; Ibrahim et al. 2024; Ye et al. 2021; Shariati et al. 2022).

## 18 Challenges Associated With the Widespread Application of Antibacterial Nanoparticles

The smart NPs that react to light, pH, and bacterial enzymes, additional challenges remain (Zhang et al. 2023). The ambiguity around cytotoxicity, oxidative stress, and the possibility of long‐term environmental buildup has limited their practical use. Xuan et al. (2023) and Wang et al. (2024) discovered several potential advancements in peptide‐loaded systems, hybrid nanoantibiotics, and CRISPR‐based carriers. Nonetheless, these advancements encounter considerable challenges, such as erratic production, elevated expenses, restricted scalability, and insufficient regulatory backing. The main objective of scientific research ought to be the development of biodegradable nanomaterials that are non‐toxic to living organisms (Xuan et al. 2023; Wang et al. 2024). Comprehensive safety evaluations, AI‐guided design, and publicly supported laws are essential for the implementation of these technologies in the real world (Olawade et al. (2024)) (Olawade et al. 2024) and Yan et al. (2020) (Yan et al. 2020). The biocompatibility of NPs is crucial for their safe use in biomedicine while mitigating potential negative effects. Their medicinal efficacy and adverse effects may be enhanced by meticulous design and surface modification (Wang et al. 2024).

## 19 Future Directions

The rise of multidrug‐resistant bacteria and other diseases has resulted in a heightened use of antibacterial nanoparticles. Future research should focus on enhancing the biocompatibility, selectivity, and environmental compatibility of these nanoparticles while reducing their toxicity (Dutt 2024). Nanoparticles need activation by certain stimuli like bacterial enzymes, pH variations, or environmental influences, such as light and magnetic fields, to function as efficient antibacterial agents. Numerous studies corroborate the notion that target‐specific nanoparticles may mitigate harmful effects. Copper sulfide (CuS) nanoparticles are used in photodynamic treatment (PDT) and photothermal therapy (PTT) to improve wound healing, and they exhibit antibacterial properties (Naskar and Kim 2022). Similarly, nanocomposite hydrogels including lignin‐based photactive nanocomposites may eradicate microbial cells upon exposure to light via a pH‐responsive controlled release mechanism. Alternatively, microbubbles with Fe3O4 nanoparticles use a magnetism‐targeted methodology (Lu et al. 2024).

According to Quiñones‐Vico et al. (2024), Pisani et al. (2024), and Costabile et al. (2024). The integration of nanoparticles with antibiotics represents a substantial advancement that amplifies therapeutic efficacy. Abdullah et al. (2024) illustrate that antibiotic‐embedded metal nanoparticles have potential effectiveness against microbial resistance. Another concern for the future is enhancing the efficacy of hybrid nano‐antibiotic systems for all stakeholders (AbdulMalek et al. 2025). Ibraheem et al. (2022) discovered that antibiotics encapsulated in polymeric nanoparticles exhibited improved bioavailability and reduced dose requirements due to sustained release and effective penetration. The beneficial carrier features of AgNPs provide them an appropriate delivery mechanism for biological pharmaceuticals. Chemically reduced AgNPs had enhanced antibacterial activity when paired with ciprofloxacin, and revealed much improved outcomes when loaded with vancomycin, as reported by Ibraheem et al. (2022) and Veriato et al. (2023). For example, nanoparticles encapsulated in cell membranes or combined with antimicrobial peptides may improve targeting effectiveness by simulating a natural defence mechanism. Nanoparticles (NPs) integrated with antimicrobial peptides or encapsulated inside cell membranes may improve targeted effectiveness by emulating the body's inherent defence systems. The unique physicochemical properties of nanoparticles may inhibit or postpone the degradation of the medication inside their encapsulation (Wang et al. 2023; Ma et al. 2022). Research indicated that the average diameter of Chitosan‐based nanostructures including Synthetic LL37 antimicrobial peptide, was 127.12 nm, with an encapsulation efficiency of around 78% (Fahimirad et al. 2021). This amalgamation of attributes generated robust antibacterial efficacy and enhanced stability. The minimal inhibitory concentrations (MIC) are 6, 23, and 31 μg/mL, respectively (Hu et al. 2021). The formulation of Nal‐P‐113‐PEG‐CSNPs (Nal‐P‐113 loaded PEG‐Chitosan nanoparticles) similarly demonstrated inhibition of the development of *Streptococcus gordonii*, *Porphyromonas gingivalis*, and *Fusobacterium nucleatu*.

### 19.1 Key Findings

This article identifies several key findings regarding the use of nanotechnology to address the global crisis of AMR as follows.

#### 19.1.1 Urgency of the AMR Crisis

Antimicrobial resistance is a significant health risk worldwide, and it directly caused nearly 1.27 million deaths in 2019. The absence of new antibiotic development and the genetic adaptability of bacteria exacerbate the crisis since it enables bacteria to respond quickly to the selection pressure.

#### 19.1.2 Unique Therapeutic Advantages of Nanoparticles

Nanoparticles (NPs) may be considered as nanobiotics that may circumvent the classical bacterial defense strategies, including efflux pumps and target alterations. This is because they are highly effective owing to their high surface‐area‐volume ratio and multivalency, which enables interactions with microbial systems to be very strong.

### 19.2 Multifaceted Antibacterial Mechanisms

#### 19.2.1 Physical Disruption

The positive charges on NPs can electrostatically interact with negatively charged bacterial surfaces, causing pitting of the cell wall, depolarization, and membrane leakage.

#### 19.2.2 Oxidative Stress

Silver (Ag), Copper (Cu), and Zinc Oxide (ZnO) are NPs that initiate the overproduction of Reactive Oxygen Species (ROS) that lead to lipid peroxidation, protein alteration, and DNA damage.

#### 19.2.3 Intracellular Interference

Small NPs are able to enter bacterial cells to block DNA replication, RNA binding, and protein translation, and eventually cause cell death.

#### 19.2.4 Synergistic Efficacy with Antibiotics

The addition of nanoparticles to the traditional antibiotics significantly enhances treatment. The methodology enables lower doses of drugs, less host toxicity, and avoids the enzymatic degradation of antibiotics. For instance, gold nanoparticles with ampicillin are capable of neutralizing β‐lactamases expressed by resistant strains such as MRSA.

### 19.3 Material‐Specific Potential

Silver (AgNPs): It is the most researched because of its strong bactericidal action with a relatively low level of toxicity, which works through the liberation of Ag+ ions and destruction of the membrane. Gold (AuNPs): They are shape‐dependent, with triangular shapes being more effective compared to spherical ones in inhibiting ATP synthase and metabolic pathways. Nitric Oxide (NO) Platforms: NO‐releasing nanoparticles show universal activity through the production of reactive nitrogen and oxygen species, which destroy microbial elements.

#### 19.3.1 Clinical and Developmental Challenges

Although promising, its use is limited due to its high cost of production and inability to be used at an industrial scale, and because of long‐term cytotoxicity and environmental accretion. Future developments should be directed toward biodegradable substances, stimuli‐responsive (e.g., pH‐ or light‐controlled release), and powerful regulation.

## 20 Conclusion

Antimicrobial resistance is becoming a growing menace in modern medicine, and it is time to stop developing new antibiotics and instead move to more multidimensional forms of intervention. Nanomedicine has been at the forefront of this change, as a platform of versatile design of so‐called nanobiotics capable of tackling the most resilient pathogens. The targeting ability of nanoparticles toward particular bacterial systems (including the physical break‐ups of biofilms and the interference with intracellular signaling) offers a powerful alternative to traditional medicines. The use of silver (Ag) and gold (Au) nanoparticles and metal oxide is the most effective because of their various mechanisms of action, which include the production of reactive oxygen species (ROS) and cell wall penetration. Moreover, the synergy between these nanomaterials and the current antibiotics not only reinstates the effectiveness of the legacy drugs but also reduces the selection pressure for additional resistance since lower, more targeted dosages can be given. This method has a lot of potential in the protection of the immunocompromised population and in enhancing the results of intensive medical modalities such as surgery and chemotherapy. Nonetheless, there are significant challenges to the large‐scale clinical use of nanotechnology. Problems with industrial scalability, excessive production costs, and concerns about long‐term cytotoxicity and environmental persistence should be mitigated by performing stringent safety tests and standard regulatory strategies. Going forward, the convergence of nanotechnology and artificial intelligence, CRISPR gene editing, and precision medicine will determine the future of AMR management. The global community could embrace the potential of nanomedicine to its fullest by encouraging multidisciplinary collaboration and guaranteeing long‐term investment in research to counter the threat of MDR bacteria and guarantee the further effectiveness of antimicrobial treatment in generations to come.

## Author Contributions

**Akmal Zubair:** conceptualization, investigation, funding acquisition, writing – original draft, visualization, writing – review and editing, validation, project administration, formal analysis. **Syeda Zaira Batool:** project administration, visualization, writing – review and editing. **Mohamed H. Helal:** validation, formal analysis. **Naila Afghan:** validation, visualization.

## Ethics Statement

The authors have nothing to report.

## Conflicts of Interest

The authors declare no conflicts of interest.

## Data Availability

Data sharing does not apply to this article as no datasets were generated or analyzed during the current study.
